# Effectiveness of self-management support interventions for people with comorbid diabetes and chronic kidney disease: a systematic review and meta-analysis

**DOI:** 10.1186/s13643-018-0748-z

**Published:** 2018-06-13

**Authors:** Edward Zimbudzi, Clement Lo, Marie L. Misso, Sanjeeva Ranasinha, Peter G. Kerr, Helena J. Teede, Sophia Zoungas

**Affiliations:** 1 Monash Centre for Health Research and Implementation, School of Public Health and Preventive Medicine, Monash University, 43-51 Kanooka Grove, Clayton, Melbourne, Victoria Australia; 2 Department of Nephrology, Monash Health, Melbourne, Victoria Australia; 3 Diabetes and Vascular Medicine Unit, Monash Health, Melbourne, Victoria Australia; 4 Department of Medicine, School of Clinical Sciences at Monash Health, Monash University, Melbourne, Victoria Australia; 5 The George Institute for Global Health, University of Sydney, Sydney, New South Wales Australia

**Keywords:** Chronic kidney disease, Diabetes, Interventions, Self-management, Systematic review, Meta-analyses

## Abstract

**Background:**

Self-management support interventions may potentially delay kidney function decline and associated complications in patients with comorbid diabetes and chronic kidney disease. However, the effectiveness of these interventions remains unclear. We investigated the effectiveness of current self-management support interventions and their specific components and elements in improving patient outcomes.

**Methods:**

Electronic databases were systematically searched from January 1, 1994, to December 19, 2017. Eligible studies were randomized controlled trials on self-management support interventions for adults with comorbid diabetes and chronic kidney disease. Primary outcomes were systolic blood pressure, diastolic blood pressure, estimated glomerular filtration rate, and glycated hemoglobin. Secondary outcomes included self-management activity, health service utilization, health-related quality of life, medication adherence, and death.

**Results:**

Of the 48 trials identified, eight studies (835 patients) were eligible. There was moderate-quality evidence that self-management support interventions improved self-management activity (standard mean difference 0.56, 95% CI 0.15 to 0.97, *p* < 0.007) compared to usual care. There was low-quality evidence that self-management support interventions reduced systolic blood pressure (mean difference − 4.26 mmHg, 95% CI − 7.81 to − 0.70, *p* = 0.02) and glycated hemoglobin (mean difference − 0.5%, 95% CI − 0.8 to − 0.1, *p* = 0.01) compared to usual care.

**Conclusions:**

Self-management support interventions may improve self-care activities, systolic blood pressure, and glycated hemoglobin in patients with comorbid diabetes and chronic kidney disease. It was not possible to determine which self-management components and elements were more effective, but interventions that utilized provider reminders, patient education, and goal setting were associated with improved outcomes. More evidence from high-quality studies is required to support future self-management programs.

**Systematic review registration:**

PROSPERO CRD42015017316.

**Electronic supplementary material:**

The online version of this article (10.1186/s13643-018-0748-z) contains supplementary material, which is available to authorized users.

## Background

The prevalence of diabetes is on the rise globally, driven primarily by the increasing incidence of type 2 diabetes in the setting of increasing overweight and obesity [1]. The International Diabetes Federation estimated that 415 million adults (aged 20–79 years) had diabetes in 2015 and 5 million deaths were attributable to diabetes and the total global health expenditure due to diabetes was 673 billion US dollars [2]. By 2040, the number of adults with diabetes (aged 20–79 years) is expected to rise to 642 million [2]. The dramatic increase in diabetes is associated with a myriad of diabetes-related complications such as cardiovascular disease, renal failure, blindness, and lower limb amputation [3].

Chronic kidney disease (CKD) is one of the commonest diabetes-related complications. Worldwide, current estimates suggests that over 500 million people have CKD, with the majority (80%) of those people living in low- and middle-income countries [4] and diabetes contributes to 30–40% of all cases of end-stage renal disease (ESRD) [5]. In developed countries, diabetes accounts for 50% of cases of treated ESRD [6]. As the prevalence of diabetes increases, the incidence of CKD is expected to increase.

Co-morbid diabetes and CKD is associated with an increased risk of a range of adverse outcomes including increased mortality [7], low health-related quality of life [8], and increased health service utilization [9]. Self-management support interventions have generated considerable interest in the management of CKD as a means of helping to improve risk factors and slow disease progression [10]. However, the effects of self-management strategies for those with co-morbid diabetes and CKD are largely unknown [11]. Many current approaches to self-management for patients with both diabetes and CKD are based on interventions for single conditions rather than for patients with complex multimorbidity [11]. Additionally, there is a huge diversity of potential self-management support interventions which have been trialed making it difficult for health care providers to select the most pragmatic and effective interventions. To date, there has been no systematic review of the literature examining the effectiveness of self-management support interventions in people with both diabetes (type 1 or type 2) and CKD.

To address this, we undertook a systematic review, which sought to answer the following questions:

1. How effective are self-management support interventions in improving patient-reported and clinical outcomes in adults with comorbid diabetes and CKD?
2. Which specific self-management components and elements are associated with improved outcomes for patients with comorbid diabetes and CKD?

## Methods

The conduct of this review was guided by the Cochrane Handbook for Systematic Reviews of Interventions [12] and conforms to the reporting guidelines of the Preferred Reporting Items for Systematic Reviews and Meta-Analyses (PRISMA) statement recommendations [13]. The protocol of this systematic review was registered on PROSPERO 2015 (registration number CRD42015017316) [14] and published [15].

### Selection criteria

Table 1 presents the Population, Intervention, Comparison, and Outcome (PICO) framework established a priori to include and exclude studies for this systematic review.

**Table 1:** Selection criteria

	Inclusion	Exclusion
Participants	Adult patients (above 18 years) with diabetes^a^ and CKD in any health care setting	Participants without the diagnosis of diabetes and CKD
Interventions	Self-management models including at least one of the following intervention components: Provider education, provider feedback, provider reminders, patient education, patient reminders, and patient financial incentives	No intervention or any intervention other than those prespecified in the inclusion criteria
Control	Clearly defined usual or standard care. This may be the chronic disease management programme that is already in place before a new model of care is introduced	Any intervention except those listed in the inclusion criteria
Outcomes	Must include at least one of the following outcomes: Primary: 1. Clinical indicators (blood pressure, eGFR, and HbA_1c_) Secondary: 1. Medication adherence 2. Self-management activity 3. Health service utilization including hospitalization 4. Health-related quality of life 5. Adverse events such as deaths	Lack of at least one relevant prespecified outcome
Study design	Randomized controlled trials and systematic reviews of randomized controlled trials	Studies reporting non-randomized studies

^a^Participants with either type 1 or type 2 diabetes were included

*CKD* chronic kidney disease which was defined as a sustained decrease in eGFR to levels less than 60 mL/min/1.73 m^2^ for a period of 3 months or longer, *eGFR* estimated glomerular filtration rate, *HbA*_*1c*_ glycated hemoglobin

### Participants

This review considered studies of people with both diabetes (type 1 or type 2) and CKD. CKD was defined as a sustained decrease in estimated glomerular filtration rate (eGFR) to levels less than 60 mL/min/1.73 m^2^ for a period of 3 months or longer [16]. In studies where the inclusion criteria were not clear, we sought clarification from the corresponding authors and such studies were excluded if we could not get verification.

### Interventions

For the purpose of this review, self-management support was defined as “the systematic provision of education and supportive interventions by health care staff to increase patients’ skills and confidence in managing their health problems, including regular assessment of progress and problems, goal setting, and problem-solving support” [17]. The core components of the interventions were provider education, provider feedback, provider reminders, patient education, patient reminders, and patient financial incentives with elements that included standardized training, multidisciplinary team, peer contact, keeping logs, goal setting skills, problem solving skills, and seeking support.

### Outcomes

Primary outcomes included clinical indicators such as blood pressure, eGFR, and HbA_1c_, and secondary outcomes included self-management activity, health service utilization, health-related quality of life (HRQOL), adherence to medications, and death.

### Study design

Randomized controlled studies (including cluster randomized controlled trials) and systematic reviews of randomized controlled studies were considered. We included English-language peer-reviewed journal articles. We excluded articles reporting non-randomized studies, narrative reviews, letters, editorials, commentaries, unpublished manuscripts, dissertations, government reports, books and book chapters, conference proceedings, meeting abstracts, lectures and addresses, and consensus development statements and guidelines.

### Literature search

We conducted a comprehensive search of literature, which has been described in detail elsewhere [15]. In brief, we identified RCTs through Medline, Medline in-process and other non-indexed citations, EMBASE, CINAHL, and all evidence-based medicine (EBM) reviews. We also searched the bibliographies of relevant studies identified by the search strategy for identification of additional studies. The databases were searched from January 1, 1994, to December 19, 2017. A detailed description of search limits is provided elsewhere (Additional file 1: Table S1). To ensure reliability, two reviewers (EZ and CL) independently scanned the titles, abstract sections, and keywords of every article obtained by the search strategy. The two reviewers retrieved full texts of potentially relevant studies and screened them independently for inclusion. During the full-text review, if the two reviewers were in doubt about the inclusion of any particular study, the third reviewer (MM) was involved. Investigators of all eligible studies were also contacted by email to request unpublished data relevant to the review.

### Data extraction and critical appraisal

Two reviewers (EZ and CL) independently extracted data relevant to the PICO framework using a specially designed data abstraction form. Information was collected on general details (title, authors, reference/source, country, year of publication, setting), participants (age, sex, inclusion/exclusion criteria, withdrawals/losses to follow-up, subgroups), results (point estimates and measures of variability, frequency counts for dichotomous variables, number of participants, intention-to-treat analysis), and validity results.

The methodological quality of each of the included studies was independently appraised by two reviewers (EZ and CL) using the Monash Centre for Health Research and Implementation (MCHRI) template [18] (Additional file 2: Table S2) and the quality of evidence using the Grading of Recommendations, Assessment, Development, and Evaluation (GRADE) approach [19]. Any disagreement was resolved by discussion with the third reviewer (MM) to reach a consensus. We contacted authors of included trials when clarification surrounding study conduct or missing data was required.

### Data synthesis and meta-analysis

Analyses of data from included trials were performed with Review Manager (RevMan version 5.3.5, The Nordic Cochrane Centre, The Cochrane Collaboration, Copenhagen, Denmark). For meta-analysis, all outcomes were continuous and results are presented as mean differences (MDs) or standard mean difference (SMD) if different scales were used [12] with 95% confidence interval (CI). A positive SMD value indicated the intervention group was superior to the control group on a positively oriented outcome measure. Data from eligible studies were pooled using the random effects model to account for heterogeneity [20]. Statistical heterogeneity was quantified using the inconsistency index-*I*^2^ statistic with “low” heterogeneity set at ≤ 25%, “moderate” 50%, and “high” ≥ 75%. To assess clinical heterogeneity, we performed a sensitivity analysis excluding a study of people with end-stage renal disease from the analysis. A subgroup analysis of pooled data based on the different self-management components was also carried out. Publication bias was not statistically assessed due to the small number of RCTs included. Statistical significance was set at *p* < 0.05 for primary and secondary outcome measures. A descriptive analysis was performed to summarize data narratively for outcomes that had unexplained heterogeneity and missing data such as means and SDs and when there was a small number of studies reporting an outcome (less than 2 studies).

## Results

### Literature search and study characteristics

**Fig. 1:**
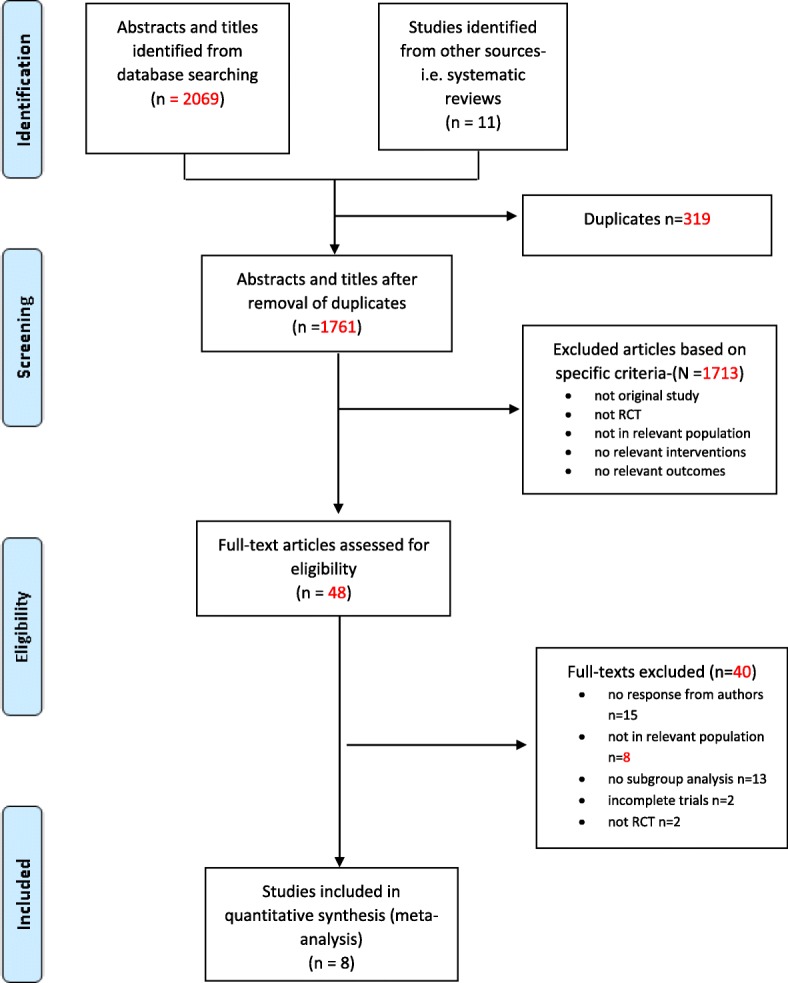
PRISMA flow diagram showing how studies were screened [13]

The results of the systematic search are shown in Fig. 1. Two thousand and eighty references were identified by the search including 11 obtained from hand-searching of reference lists of seven systematic reviews [21–27] obtained from the search. After removal of duplicates and screening of titles and abstracts, 48 full-text articles were reviewed for further assessment. Following the full-text review, 40 articles were excluded based on reasons outlined in Fig. 1 and Additional file 3: Table S3. Eight studies [28–34] remained and were included in the systematic review. One of the studies (the SURE study) [30] had a duplicate publication [9], which reported on cost implication of the intervention. We treated the two publications as one study.

Characteristics of the eight included studies are presented in Table 2. Three studies were performed in the UK [28, 31, 34] and one each in Canada [29], China [30], USA [32], Netherlands [33], and Australia [35]. Four studies [28, 29, 31, 33] were conducted in a primary care setting, two in hospital-based outpatient clinics [34, 35], one in hospital [30], and one in hemodialysis or peritoneal dialysis units [32]. Three studies [30, 33, 34] included patients with type 2 diabetes only; two studies [29, 32] specified having patients with both type 1 and type 2, and three studies [28, 31, 35] did not specify the type of diabetes. There was a substantial variation in the study sample sizes (*n* = 28 to 205), interventions, and follow-up period (3 to 24 months). Most of the studies were excluded due to inadequate data reported and no responses from authors (*N* = 15) and lack of evidence demonstrating that they included the correct population relevant to this review (*N* = 13) (Additional file 3: Table S3).

**Table 2:** Characteristics of included studies

Study/setting	*N* ^a^	Population	Intervention (content, delivery and duration characteristics)	Control	Outcomes^b^	Follow-up/dropouts/sample size analyzed	Risk of bias
Blakeman et al.2014^a^[28] Primary care, 24 general practices in UK	*N* = 101 IG—49 CG—52 ND by gender	Adult patients who had a diagnosis of stage 3 CKD. Type of diabetes not specified.	Information and telephone-guided access to community support. The intervention entailed provision of: 1. A kidney information guidebook. 2. A PLANS booklet and access to an interactive website with tailored access to local resources. 3. Telephone support from a peer support worker. The intervention was delivered for 4 weeks.	Patients were provided with the guidebook and website link at the end of the trial.	Self-management, blood pressure control, and HRQOL	Follow-up: 6 months Dropouts: ND Sample size analyzed: *n* = 101 IG: *n* = 49 CG: *n* = 52	Moderate
Barrett et al. 2011^a^ [29] Primary care, 5 urban centers in Canada	*N* = 149 IG—73 CG—76 ND by gender	40–75 years with CKD, eGFR between 25 and 60 mL/min per 1.73m^2^ Type 1 and 2 diabetes	Nurse-coordinated care focused on risk factor modification. Intervention group participants had additional clinical care delivered by the study nurse and nephrologist guided by protocols aimed at achieving the prespecified targets but focused on the needs of the individual. Study visits occurred every 4 months for the duration of the study.	Patients received usual care that their health care providers felt indicated.	HbA_1c_, blood pressure, and eGFR	Follow-up: 24 months Dropouts: ND Sample size analyzed: *n* = 149 IG: *n* = 73 CG: *n* = 76	Moderate
Chan et al. 2009 [30] Hospitals, 9 public hospitals in China	*N* = 205 Male: IG—66 CG—67 Female: IG—38 CG—34	Type 2 diabetic patients with renal insufficiency	Structured care managed by a diabetes team. A dietitian saw the intervention group after randomization. ACE inhibitor or ARB therapy was started in treatment-naive patients with monitoring of renal function at week 2, then every 4 weeks for 12 weeks and subsequently every 8–12 weeks, throughout the study period. A doctor-nurse team saw patients every 3 months and more often if indicated.	Patients were managed according to the usual clinic practice as defined by the respective hospital with no modification.	Blood pressure, HbA_1c_, eGFR, and death	Follow-up: 24 months Dropout: *n* = 38 IG: *n* = 20 CG: *n* = 18 Sample size analyzed: *n* = 167 IG: *n* = 84 CG: *n* = 83	Low
McManus et al. 2014^a^ [31] Primary care practices in UK	*N* = 28 I—10 C—18 ND by gender	> 35 years with stroke, CHD, diabetes, or CKD and hypertension Type of diabetes not specified.	Self-monitoring of blood pressure and individualized self-titration algorithm. Patients in the intervention group were trained to self-monitor blood pressure in 2 or 3 sessions, each lasting approximately an hour. Following training, intervention patients went to their family physician to agree with the individualized 3-step plan to increase or add antihypertensive medications. The intervention occurred for the duration of the study.	Patients had routine blood pressure check and medication review with the participating family physician.	Blood pressure	Follow-up: 6 and 12 months Dropout: ND Sample size analyzed: *n* = 28 IG: *n* = 10 CG: *n* = 18	Moderate
McMurray et al. 2002 [32] Hemodialysis or peritoneal dialysis units in USA	*N* = 83 Male: IG—24 CG—21 Female: IG—21 CG—17	ESRD on either HD or PD with a diagnosis of a type 1 or type 2 diabetes mellitus	Diabetes education and care management program. The diabetes care manager delivered self-management education, diabetes care monitoring and management, and motivational coaching to the intervention group. The renal dietitian performed initial nutritional counseling. Follow-up was performed at hemodialysis sessions or monthly for peritoneal dialysis patients.	Patients received standard diabetes care prevalent at the dialysis facility as directed by their physician.	HbA_1c_, HRQOL, self-management behavior, and hospitalization	Follow-up: 12 months Dropout: *n* = 0 Sample size analyzed: *n* = 83 IG: *n* = 45 CG: *n* = 38	High
Scherpbier-de Haan et al. 2013^a^ [33] Primary care, 9 general practices in Netherlands	*N* = 65 Male: IG—17 CG—16 Female: IG—22 CG—10	> 18 years, hypertension or type 2 diabetes mellitus, and eGFR of < 60 mL/min/1.73m^2^	Shared care. The nurse practitioner saw patients every 3 months for a 20-min consultation, in which blood pressure treatment was the main aim. Patients and nurse practitioners decided together which other treatment goals were to be prioritized. GPs supervised the consultation afterwards. GPs and nurse practitioners could, if necessary, consult a nephrology team in a protected digital environment.	No intervention other than routine review.	Blood pressure, eGFR, and HbA_1c_	Follow-up: 12 months Dropout: ND Sample size analyzed: *n* = 65 IG: *n* = 39 CG: *n* = 26	High
Steed et al. 2005 [34] Outpatient clinics at two inner city hospitals in UK	*N* = 124 Male: IG—44 CG—44 Female: IG—21 CG—15	Type 2 diabetes, with renal insufficiency	The University College London-Diabetes Self-management Programme (UCL-DSMP) The intervention was a group-based program consisting of five 2.5-h sessions held weekly for 5 weeks, plus one booster session of 2.5 h held 3 months after the end of the intervention. Facilitators were diabetes specialist nurses and dieticians.	No intervention other than completion of assessments.	HbA_1c_, self-management practices, and HRQOL	Follow-up: 3 months Dropout: *n* = 10 IG: *n* = 10 CG: *n* = ND Sample size analyzed: *n* = 114 IG: *n* = 55 CG: *n* = 59	High
Williams et al. 2012^a^ [35] Outpatient clinics in Australia	*N* = 80 Male: IG—22 CG—23 Female: IG—17 CG—18	Aged > 18 years with diabetes, CKD, and systolic hypertension Type of diabetes not specified.	Multifactorial Medication Self-Management Intervention (MESMI) 1. Self-monitoring of blood pressure. 2. An individualized medication review. 3. A 20-min digital versatile disc (DVD) 4. Fortnightly motivational interviewing follow-up telephone contact for 12 weeks to support blood pressure control and optimal medication self-management. A renal nurse trained in motivational interviewing delivered all components of the intervention.	No intervention	Blood pressure, HbA_1c_, eGFR	Follow-up: 3, 6, and 12 months Dropout: *n* = 5 IG: *n* = 3 CG: *n* = 2 Sample size analyzed: *n* = 75 IG: *n* = 36 CG: *n* = 39	Low

^a^These are participants who had diabetes and chronic kidney disease from the included studies. Additional data obtained from corresponding authors

^b^Outcomes relevant to this systematic review; total *N* = 835

*CKD* chronic kidney disease, *CHD* coronary heart disease, *HRQOL* health-related quality of life, *eGFR* estimated glomerular filtration rate, *HbA*_*1c*_ glycated hemoglobin, *ESRD* end-stage renal disease, *HD* hemodialysis, *PD* peritoneal dialysis, *IG* intervention group, *CG* control group, *ND* no data available, *ACE* angiotensin-converting enzyme inhibitors, *ARB* angiotensin II receptor blockers, *PLANS* patient-led assessment for network support

### Elements and components of self-management support interventions

**Fig. 2:**
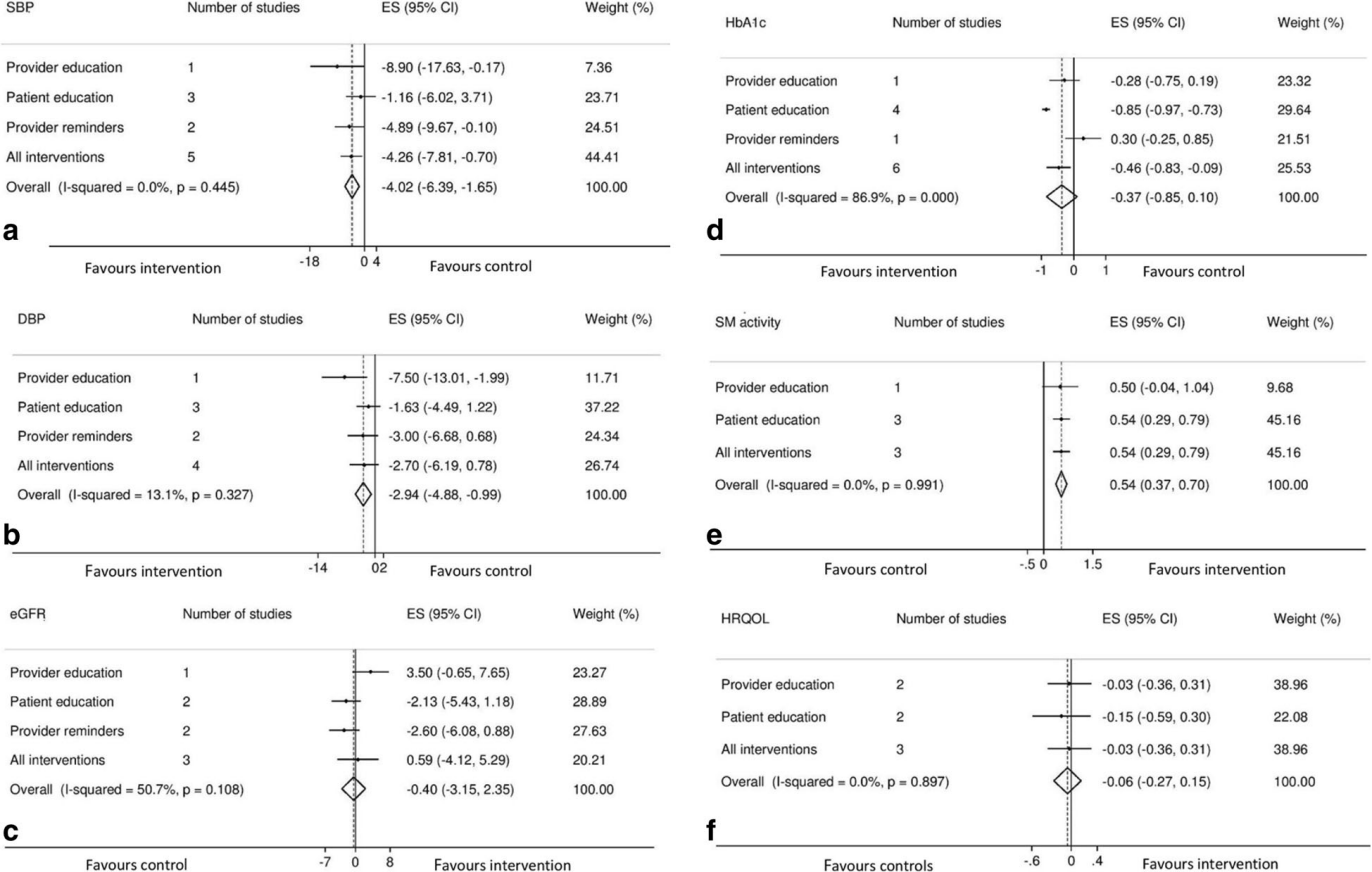
Meta-analyses showing effect of the different intervention components on **a** systolic blood pressure, **b** diastolic blood pressure, **c** estimated glomerular filtration rate, **d** glycated hemoglobin (%), **e** self-management activity, and **f** health-related quality of life. Intervention components with one trial are not based on meta-analysis (individual trial result is presented)

All the included studies had a theoretical underpinning for their self-management elements. Key elements of these interventions were derived from the Chronic Care Model [36], the Stanford Model [37], the Expert Patient Programme [38], and the Flinders Model [39] (Table 3). These elements include standardized training, multidisciplinary team, peer contact, keeping logs, goal setting and problem solving skills, and seeking support. There was a marked variation in the elements of interventions in terms of both content and delivery (Tables 2 and 3). Seven studies [28–34] described interventions underpinned by care coordination and a team based-approach with a focus on patient self-management and working collaboratively. The intervention components reported were patient education [28, 30–32, 34, 35], provider reminders [29, 30], and provider education [28, 33] (Fig. 2).

**Table 3:** Key elements to effective planned self-management support interventions

Study	Standardized training	Multidisciplinary team	Peer contact	Keeping logs	Goal setting skills	Problem solving skills	Seeking support
Blakeman et al. [28]	*	*	*		*		*
Barrett et al. [29]		*					
Chan et al. [30]	*	*					
McManus et al. [31]	*	*		*	*	*	*
McMurray et al. [32]	*	*			*	*	
Scherpbier-de Haan et al. [33]	*	*			*		*
Steed et al. [34]	*	*	*		*		
Williams et al. [35]	*			*	*		*

The studies utilized elements derived from the following self-management models: (a) the Chronic Care Model, (b) the Stanford Model, (c) the Expert Patient Programme, and (d) the Flinders Models

*means respective self-management element was used by the study

### Delivery characteristics

The delivery characteristics for the interventions are shown in Table 2. The study duration ranged from 3 to 24 months, with two studies having a duration of less than 12 months. The potential influence of follow-up duration on the estimates was explored by plotting the effect size against follow-up time, and there was no relationship between the two. Most of the studies had more than one delivery element. Five studies utilized face-to-face delivery [30–32, 34, 35], three had the self-management component delivered by telephone [28, 30, 35], and four used written information, websites, and protocols [28, 29, 31, 33] to guide the delivery of the interventions. All studies apart from one [35] had members of the multidisciplinary team facilitating the delivery of self-management support interventions. The members included nurses, dietitians, social workers, general practitioners, diabetologists, endocrine trainees, and nephrologists.

### Risk of bias in included studies

Additional file 4: Figure S1 and Additional file 5: Figure S2 present an overview of the risk of bias for the included studies assessed against six risk-of-bias criteria which included selection (randomization and allocation), performance, detection, attrition, and reporting bias. Five studies [28–31, 35] reported random sequence generation, and four studies [28–30, 35] demonstrated adequate allocation concealment. The majority of studies had high risk of performance bias [28, 29, 31, 32, 34] and detection bias [28, 29, 31, 32, 34]. Only one study had a low risk of performance bias [33] and one study a low risk of detection bias [35]. Seven studies [28, 30–35] had a low risk of attrition bias, and all included studies had a low risk of reporting bias.

### Effects of interventions

Table 4 provides the main comparison between groups, which had self-management support interventions, and controls. The study interventions were of varying intensity levels. Meta-analyses were only performed for systolic blood pressure, diastolic blood pressure, eGFR, HbA_1c_, diabetes self-management activity, and HRQOL.

**Table 4:** Summary of findings for the main comparison

Self-management compared with control for participants with diabetes and chronic kidney disease				
Patient or population: patients with diabetes and chronic kidney disease Settings: community, primary care, hospital outpatient Intervention: self-management Comparison: standard care				
Outcomes	Impact	Relative effect estimate (95% CI)	No. of studies (participants)	Quality of evidence (GRADE)^a^
Systolic blood pressure Follow-up: 6 to 24 months [28–31, 33, 35]	SBP MDs ranged from − 8.90 to 3.60 mmHg.One study* [28] was excluded from the meta-analysis due to insufficient data.	MD − 4.26 (− 7.81, − 0.71)	6 (577)	Low^1^
Diastolic blood pressure Follow-up: 12 to 24 months [30, 31, 33, 35]	DBP MDs − 7.50 to 2.30 mmHg	MD − 2.70 (− 6.19, 0.78)	4 (336)	Low^1^
eGFR Follow-up: 12 to 24 months [29, 30, 33, 35]	Estimated GFR MDs ranged from -2.60 to 3.50 mL/min/1.73 m^2^. One study* [29] was excluded from the meta-analysis due to insufficient data.	MD 0.59 (− 4.12, 5.29)	4 (499)	Very low^1, 2, 3^
HbA_1c_ Follow-up: 3–24 months [29, 30, 32–35]	HbA_1c_ MDs ranged from − 0.90 to 0.30%.	MD − 0.46% (− 0.83, − 0.09)	6 (595)	Low^1, 3^
Adherence to medications Follow-up: 12 months [35]	One study [35] identified no difference in medication adherence between the control and intervention groups using the Morisky scale.	Not estimable	1 (80)	Moderate^4^
Self-management activity Follow-up: 3–12 months [28, 32, 34]	The self-management SMDs for the three studies ranged from 0.31 to 0.99.	SMD 0.56 (0.15, 0.97)	3 (308)	Moderate^5^
Health service utilization Follow-up: 6–24 months [28, 30, 32]	Two studies [28, 30] showed no differences in hospitalization between the intervention and control groups and one study [32] reported that the study group had lower hospitalization rates.	Not estimable	3 (389)	Low^1^
Health-related quality of life Follow-up: 3–12 months [28, 32–34]	Two studies [28, 33] showed no difference in quality of life between the intervention and control groups, and in the other two studies [32, 34], the intervention group showed a statistically significant improvement in the quality of life assessment.	SMD − 0.03 (− 0.36, 0.31)	4 (373)	Moderate^1^
Death Follow-up: 12 to 24 months [30–32]	The three studies showed no differences in mortality between the intervention and control groups.	Not estimable	3 (354)	Very low^1, 6^

High quality: further research is very unlikely to change our confidence in the estimate of effect. Moderate quality: further research is likely to have an important impact on our confidence in the estimate of effect and may change the estimate. Low quality: further research is very likely to have an important impact on our confidence in the estimate of effect and is likely to change the estimate. Very low quality: we are very uncertain about the estimate

*SBP* systolic blood pressure, *MDs* mean differences, *CI* confidence interval, *DBP* diastolic blood pressure, *eGFR* estimated glomerular filtration rate, *HbA*_*1c*_ glycated hemoglobin, *SMD* standard mean difference

^a^Studies were excluded from the meta-analysis due to non-availability of data. GRADE Working Group grades of evidence

^1^The majority of the studies were not blinded to patients or outcome assessors and they did not report allocation concealment. The quality of evidence was downgraded by 2

^2^There was a considerable degree of inconsistency with several studies reporting effects in opposite directions. The quality of evidence was downgraded by 1

^3^One study reported on eGFR, but there was no data

^4^Relative estimate was not estimable. There were some discrepancies in responses as participants reported that they had no problem remembering to take their medications but at the same time they forgot to take their medications and vice versa. This study had allocation concealment and was blinded to investigators and outcome assessors. We did not downgrade based on limitations

^5^Heterogeneity was moderate (*I*^2^ = 63%). The 95% confidence intervals for some individual studies were narrower

^6^Death was reported by three studies (for the subgroup of patients with diabetes and chronic kidney disease), but the relative effect was not estimable

### Primary outcomes

#### Systolic blood pressure

**Fig. 3:**
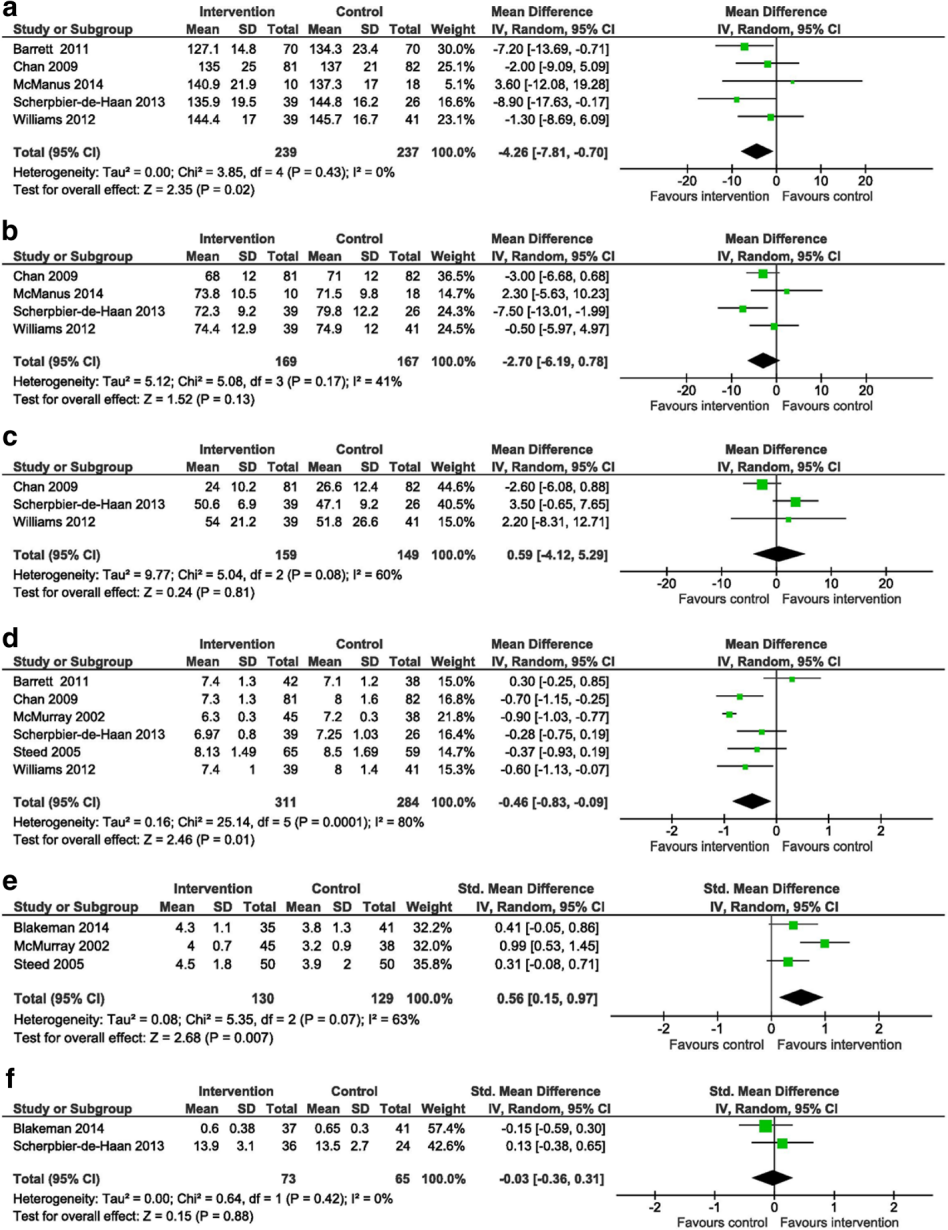
Forest plots displaying the effectiveness of self-management support interventions in improving outcomes for patients with diabetes and chronic kidney disease: **a** systolic blood pressure, **b** diastolic blood pressure, **c** estimated glomerular filtration rate, **d** hemoglobin A_1c_, **e** self-management activity, and **f** health-related quality of life. The *x*-axis represents mean differences or standard mean differences. The 95% confidence intervals (CI) for individual studies are represented by a horizontal line and by a diamond for pooled effect. SD standard deviation, IV inverse variance

Treatment effects for systolic blood pressure were reported by six studies [28–31, 33, 35] with mean systolic blood pressures ranging from 127 to 144 mmHg for the intervention groups and 134 to 146 mmHg for the control groups. Two of the six studies [29, 33] utilizing structured care and shared care as interventions reported significant improvements in blood pressure in the intervention groups compared to the control groups. Barrett et al. [29] reported a mean difference (MD) of − 7.20 mmHg (95% CI − 13.69 to 0.71, *p* < 0.05) between the intervention and control groups, while the study by Scherpbier-de Haan et al. [33] showed a MD of − 8.90 mmHg (17.63 to − 0.17, *p* < 0.05) (Fig. 3a). Data was pooled from five studies [29–31, 33, 35], which were deemed sufficiently homogenous to conduct a meta-analysis. The intervention group had a significantly lower systolic blood pressure than the control group [Fig. 3a; MD − 4.26 mmHg (95% CI − 7.81 to − 0.70) *p* = 0.02].

#### Diastolic blood pressure

Four studies [30, 31, 33, 35] reported mean diastolic blood pressures ranging from 68 to 74 mmHg for the intervention groups and 71 to 80 mmHg for the control groups. Significantly lower diastolic blood pressures were reported in two studies by Chan et al. [30] and Scherpbier-de Haan et al. [33]: MDs in diastolic blood pressure of − 3 mmHg (95% CI − 6.68 to 0.68) and − 7.5 mmHg (95% CI − 13.01 to − 1.99) respectively (Fig. 3b). Data from four studies was available for a meta-analysis. There was no significant difference in the diastolic blood pressure of the intervention and control groups [Fig. 3b; MD − 2.70 (95% CI − 6.19 to 0.78) *p* = 0.13].

#### Estimated glomerular filtration rate

Estimated glomerular filtration rate was evaluated by four studies [29, 30, 33, 35]. Data from three [30, 33, 35] studies were available for a meta-analysis. The mean differences for eGFR among the three studies ranged from − 2.6 to 3.5 mL/min/1.73 m^2^. There was no significant difference in the eGFR of the intervention and control groups [Fig. 3c; MD − 0.59 (95% CI − 4.12 to 5.29) *p* = 0.81]. However, a moderate degree of heterogeneity was detected (*I*^2^ = 60%).

#### Hemoglobin A_1c_

Six studies [29, 30, 32–35] reported mean HbA_1c_ levels ranging from 6.3 to 8.1% for the intervention groups and 7.1 to 8.5% for the control groups. Three studies [30, 32, 35] which included structured care managed by a diabetes team, diabetes education and care management program, and multifactorial medication self-management reported lower HbA_1c_ levels in the intervention groups (MDs ranging from − 0.90 to − 0.60%) than the control groups (Fig. 3d). In one study [29], which utilized the nurse-coordinated care intervention, there was a similar increase in the proportion of patients meeting HbA_1c_ targets in both the intervention and control groups. Data from the six studies were available for a meta-analysis. The intervention group had significantly lower HbA_1c_ levels than the control group [Fig. 3d; MD of − 0.5% (95% CI − 0.8 to − 0.1) *p* = 0.01]. However, a high degree of heterogeneity was detected (*I*^2^ = 80%, *p* = 0.0001). A sensitivity analysis excluding the study with patients who had ESRD [32] confirmed that the intervention group had significantly lower HbA_1c_ levels than the control group [MD of − 0.3% (95% CI − 0.68 to − 0.01) *p* = 0.04].

### Secondary outcomes

#### Self-management activity

Three studies [28, 32, 34] assessed self-management activity and reported significant improvements in most self-management activities evaluated. Two studies utilized the Summary of Diabetes Self-Care Activity [28, 34] questionnaire, while one used the Diabetes Self-Care Knowledge questionnaire and Diabetes Self-Care Behaviour Inventory [32]. The SMD in self-care for the three studies ranged from 0.31 to 0.99. Data from all three studies were included in a meta-analysis. There was a significant increase in self-care activities in the intervention groups compared to the control groups [Fig. 3e; SMD of 0.56 (95% CI 0.15 to 0.97) *p* = 0.007]. However, a moderate degree of heterogeneity was detected (*I*^2^ = 63%, *p* = 0.07). A sensitivity analysis excluding the study with patients who had ESRD [32] showed significant improvements in most self-management activities evaluated [SMD of 0.35 (95% CI 0.06 to 0.65) *p* = 0.02].

#### Health service utilization

Three studies [28, 30, 32] evaluated the effect of self-management support interventions on health service utilization. Chan et al. [30] reported similar rates of clinical events, hospitalization, and emergency room visits. Among the nine study sites, the structured care group reported lower event rates than the usual care group in five hospitals, higher event rates than the usual care group in two hospitals, and similar event rates in two hospitals. After a 2-year period, the structured care group were more likely to achieve three or more treatment goals [61% (*n* = 63) vs. 28% (*n* = 28)] and those who attained three or more treatment goals (*n* = 91) had a 60% lower risk of the primary end point (death and/or renal end point creatinine > 500 μmol/L or dialysis) compared with those who did not attain three or more treatment goals (*n* = 114) [14 vs. 34; RR 0.43 (95% CI 0.21 to 0.86)]. Blakeman et al. [28] reported a mean (SD) service use of 7.6 (7.7) and 6.1 (3.6) for the intervention and control groups respectively (*p* = 0.27). McMurray et al. [32] reported a significant progression in diabetic-related peripheral vascular/neuropathic disease in the control group from a baseline score of 2.7 to a 12-month foot risk assessment score of 3.3, whereas the study group did not show this progression (*p* < 0.02). The intervention group also had a statistically significant lower hospitalization rate for diabetes, peripheral vascular disease, infection, and amputation-related admissions (*p* < 0.05).

#### Health-related quality of life

Four studies examined health-related quality of life [28, 32–34]. Two studies [32, 34] which had missing summary data were not included in the meta-analysis. There was no significant difference in HRQOL scores between the intervention and control groups [Fig. 3f; SMD of − 0.03 (95% CI − 0.36 to 0.31) *p* = 0.88]. A low degree of heterogeneity was detected (*I*^2^ = 0%, *p* = 0.42). All the four studies used different instruments for measuring HRQOL. Blakeman et al. [28] measured HRQOL with the EuroQol five dimensions questionnaire (EQ-5D) and reported no significant difference in mean (SD) EQ5D scores in the intervention and control groups respectively (*p* = 0.52). Steed et al. [34] showed differences in diabetes specific quality of life as measured by the Audit of Diabetes-Dependent Quality of Life (ADDQoL) questionnaire (*p* < 0.01). McMurray et al. [32] evaluated patient quality of life using a questionnaire adapted from the standardized Diabetes Form 2.1 and found that the intervention group had significant improvement in the quality-of-life assessment category of diabetes symptoms (*p* < 0.001). Scherpbier-de Haan et al. [33] reported no significant difference in mean (SD) WONCA scores in the intervention and control groups respectively (*p* = 0.40).

#### Medication adherence

Medication adherence was assessed in one study [35], which reported no difference in medication adherence between groups using pill counts. The mean adherence rate to the medications at the completion of the study was 66% in the control group and 58.4% in the intervention group (*p* = 0.16).

#### Death

Three studies [30–32] reported on death. Chan et al. [30] reported eight deaths in the structured care group (*N* = 104) and 11 in the usual care group (*N* = 101). In a study by McManus et al. [31], one patient died in each group and neither death was study-related. McMurray et al. reported no difference in mortality between the control and intervention groups.

## Discussion

In this systematic review of eight studies among 835 patients with comorbid diabetes and CKD, there was moderate-quality evidence that self-management support interventions significantly improved self-management activity compared to usual care and low-quality evidence that these interventions significantly improved HbA_1c_ and systolic blood pressure but not diastolic blood pressure, eGFR, and HRQOL. The self-management components that were effective across these outcomes included provider reminders, patient education, and goal setting provided in multidisciplinary settings. In addition, treatment effects could not be quantitatively estimated for medication adherence, health service utilization, and death due to marked heterogeneity and insufficient data.

Our findings suggest that provider reminders, patient education, and goal setting may be associated with improved systolic blood pressure, HbA_1c_, and self-management activity. This is consistent with results from other studies among patients with hypertension [40] and type 2 diabetes mellitus [41]. Goal setting, reported in three studies [28, 32, 34], appeared to be an important self-management element to enhance self-care. This supports evidence from a previous study among patients with diabetes [42], which has suggested that a goal setting intervention along with a diabetes self-management guide help patients set and achieve healthy behavioral goals.

Although we found statistically significant increases in self-management activity with the self-management support interventions studied, the clinical relevance of these effects must be considered. A SMD of 0.5 has previously been reported as likely to represent a meaningful change or a minimal important difference in patient-reported outcomes [43, 44]. Our pooled estimate of 0.56 SMD units (range 0.15 to 0.97) thus suggests that an appreciable number of patients with diabetes and CKD may benefit from the self-management support interventions studied.

The Chronic Care Model (CCM) [45] provides a useful framework which explains how the multidisciplinary setting drives behaviour change especially for patients with complex diseases who require multi-faceted approaches to care. The benefits of the CCM include improved clinical outcomes [46–48], patient empowerment, and education [49]. Components of self-management support have been shown to be particularly effective when delivered by a multidisciplinary team for patients with CKD [50]. The reasons for this are that multidisciplinary members bring self-management expertise and they provide opportunities for further self-management support. In support of this, the KDIGO guidelines suggest that people with progressive CKD should be managed in a multidisciplinary care setting [51]. In this review, we cannot fully ascertain whether multidisciplinary settings led to the effectiveness of self-management support interventions since all included studies consisted of multidisciplinary teams.

These findings need to be considered in light of the very low to moderate quality of evidence examined. Reasons include potential biases in the methodological conduct of studies (including challenges in blinding investigators, participants, and outcome assessors in behavioral intervention studies [52]) and the small numbers of studies per outcome which limited interpretation of efficacy for the specific self-management support interventions investigated. There was marked heterogeneity especially for studies that reported on eGFR, HbA_1c_, and self-management activity. The reasons for this could be (1) the size of the included studies (small studies have been shown to be more heterogeneous than larger studies [53]) and (2) the variability related to the quality of the studies, characteristics of enrolled participants, and administered interventions. Our results could have also been biased by the exclusion of 28 studies due to non-response from corresponding authors and failure to specify subgroup analysis. Additionally, some studies compared interventions with usual care, which included key intervention components such as patient education, and specialist consult that could not be withheld due to ethical concerns [29, 30]. Consequently, these biases may have weakened the effects of self-management support interventions on outcomes.

The review has a number of strengths. Firstly, to our knowledge, this is the first comprehensive review of evidence on self-management support interventions for patients with both diabetes and CKD. Secondly, this review is underpinned by the use of reliable tools, a peer-reviewed and published protocol, and rigorous methods that included efforts to retrieve additional methods, information, and data from study authors to ensure that accurate data were included and synthesized.

The review had a number of limitations. We excluded studies published in languages other than English. Another limitation was the assumption that self-management support interventions were standardized when practically many aspects of self-management, particularly those delivered outside the health care setting, are not. Therefore, we relied on subjective judgment to include or exclude studies when self-management support interventions were not explicitly stated. There was also considerable threat to internal validity due to the low quality of evidence from included studies stemming from difficulties in blinding of behavioral interventions [52]. Lastly, the interpretation of results from this review should take into consideration marked variation in self-management support interventions and outcome measures in the included studies.

Findings from this review have several implications to research and practice. First, a gap of research focusing on diabetes self-management support interventions and outcomes for patients with comorbid diabetes and CKD has been highlighted. Future research should therefore focus on studies designed primarily for people with both diabetes and CKD, and when a study among people with other chronic diseases includes this sub-population, a consistent approach to the conduct and reporting of secondary analysis should be rigorously followed. Second, there should be standardization of outcome measures such as HRQOL to reduce between-study heterogeneity and more studies should measure hard clinical end points and patient-reported outcomes like medication adherence. Additionally, we have shown that self-management support interventions may improve outcomes for people with comorbid diabetes and CKD, but the effect of these interventions beyond 24 months and the intensity of the interventions required still need to be explored. Well-designed longitudinal studies that compare the components of multifaceted interventions are required to understand which components are essential for producing beneficial effects. Such studies may also gather data essential for the development of a complex RCT that can test self-management as an intervention.

## Conclusion

Self-management support interventions may improve self-care activities, systolic blood pressure, and HbA_1c_ in patients with comorbid diabetes and CKD. This evidence is based on low to moderate quality studies with relatively few study participants. It was not possible to determine which self-management support components and elements were more effective, but interventions that utilized provider reminders, patient education, and goal setting provided in multidisciplinary settings were associated with improved outcomes. More evidence from high-quality studies is required to support future self-management programs.

## Additional files

Additional file 1: **Table S1.** Ovid MEDLINE search strategy conducted on 19 December 2017. (DOCX 17 kb)

Additional file 2: **Table S2.** Template for critical appraisal of a randomized controlled trial. (DOCX 20 kb)

Additional file 3: **Table S3.** Characteristics of excluded studies (ordered alphabetically). (DOCX 12 kb)

Additional file 4: **Figure S1.** Risk of bias: review authors' judgements about each risk of bias item presented as percentages across all included studies. (PNG 205 kb)

Additional file 5: **Figure S2.** Methodological quality summary: review authors' judgements about each methodological quality item for each included study. (PNG 583 kb)

## Abbreviations

ADDQoL: Audit of Diabetes-Dependent Quality of Life
CCM: Chronic Care Model
CI: Confidence intervals
CINAHL: Cumulative Index to Nursing and Allied Health Literature
CKD: Chronic kidney disease
EBM: Evidence-based medicine
eGFR: Estimated glomerular filtration rate
EMBASE: Excerpta Medica database
EQ5D: European Quality of Life-5 Dimensions questionnaire
ESRD: End-stage renal disease
GRADE: Grading of Recommendations, Assessment, Development, and Evaluation
HbA_1c_: Glycated hemoglobin
HRQOL: Health-related quality of life
MCHRI: Monash Centre for Health Research and Implementation
MD: Mean difference
PICO: Population, Intervention, Comparison, and Outcome
PRISMA: Preferred Reporting Items for Systematic Reviews and Meta-Analyses
RCTs: Randomized controlled trials
SDSCA: Summary of Diabetes Self-Care Activity questionnaire
SMD: Standard mean difference
SURE study: Structured versus usual care on renal endpoint in type 2 diabetes
WONCA scores: World Organization of National Colleges, Academies, and Academic Associations of General Practitioners/Family Physicians
