# Supplementary material for: Effectiveness of self-management support interventions for people with comorbid diabetes and chronic kidney disease: a systematic review and meta-analysis
Source: Syst Rev. 2018 Jun 13;7:84. doi: 10.1186/s13643-018-0748-z (PMC6001117; doi:10.1186/s13643-018-0748-z)
Supplement: Supplementary file 1 — Table S1. Ovid MEDLINE search strategy conducted on 19 December 2017. (DOCX 17 kb) [file 13643_2018_748_MOESM1_ESM.docx]

**Table S1. Ovid Med-line search strategy conducted on 19 December 2017**

| 1 | exp Chronic Disease/ | 265 711 |
| --- | --- | --- |
| 2 | (chronic adj3 (illness* or disease* or condition*)).mp. | 478 794 |
| 3 | chronic disease [therapy.mp](http://therapy.mp/). | 91 |
| 4 | or/1-3 | 478 794 |
| 5 | kidney diseases/ or anuria/ or diabetic nephropathies/ or hypertension, renal/ or hypertension, renovascular/ or renal insufficiency, chronic/ | 149 471 |
| 6 | chronic kidney [disease.mp](http://disease.mp/). | 35 707 |
| 7 | (chronic kidney or chronic renal).mp. | 69 323 |
| 8 | (CKD or CRD).mp. | 22 382 |
| 9 | diabetes mellitus/ or diabetes mellitus, type 1/ or wolfram syndrome/ or diabetes mellitus, type 2/ or diabetes mellitus, lipoatrophic/ or diabetic ketoacidosis/ or donohue syndrome/ | 302 724 |
| 10 | (MODY or NIDDM or T2DM or T2D).mp. | 25 653 |
| 11 | (non insulin* depend* or noninsulin* depend* or noninsulin?depend* or non insulin?depend*).mp. | 13 377 |
| 12 | ((typ? 2 or typ? II or typ?2 or typ?II) adj3 diabet*).mp. | 155 407 |
| 13 | (((late or adult* or matur* or slow or stabl*) adj3 onset) and diabet*).mp. | 4 327 |
| 14 | (IDDM or T1DM or T1D).mp. | 14 813 |
| 15 | (insulin* depend* or insulin?depend*).mp. | 31 751 |
| 16 | ((typ? 1 or typ? I or typ?1 or typ?I) adj3 diabet*).mp. | 87 507 |
| 17 | (insulin* defic* adj2 absolut*).mp. | 103 |
| 18 | or/5-17 | 520 728 |
| 19 | exp Consumer Participation/ | 40 341 |
| 20 | exp Self Care/ | 53 884 |
| 21 | exp Self Concept/ | 105 711 |
| 22 | ((self or self directed or self-directed or self monitor* or self-monitor* or symptom*) adj (care or help or manag* or efficacy or admin* or concept)).mp. | 181 439 |
| 23 | patient financial [incentives.mp](http://incentives.mp/). | 12 |
| 24 | health education/ or consumer health information/ or health literacy/ or patient education as topic/ | 149 039 |
| 25 | Health Communication/ | 1 536 |
| 26 | interdisciplinary communication/ | 16 493 |
| 27 | ((consumer or patient*) adj2 (educat* or information or particip* or behavio?r*)).mp. | 196 797 |
| 28 | ((health educat* or health information) adj2 (program* or intervention* or meeting* or session* or strategy* or workshop* or visit* or method* or material* orcampaign*)).mp. | 5 505 |
| 29 | access to [expertise.mp](http://expertise.mp/). | 51 |
| 30 | availability of clinical [information.mp](http://information.mp/). | 24 |
| 31 | Reminder Systems/ | 3 309 |
| 32 | patient [reminders.mp](http://reminders.mp/). | 98 |
| 33 | Pamphlets/ | 3 860 |
| 34 | (leaflet* or booklet* or poster* or pamphlet*).mp. | 301 903 |
| 35 | ((written or printed or oral) adj information).mp. | 1 896 |
| 36 | (provider adj2 (educat* or feedback or remind* or behavio?r)).mp. | 1 448 |
| 37 | Health Care Reform/ | 32 276 |
| 38 | health care [reform.mp](http://reform.mp/). | 34 456 |
| 39 | exp Patient Care Management/ | 731 770 |
| 40 | (care co-ordinat* or care coordinat*).mp. | 2 869 |
| 41 | chronic disease management [model.mp](http://model.mp/). | 42 |
| 42 | exp "Continuity of Patient Care"/ | 228 607 |
| 43 | continuity of patient [care.mp](http://care.mp/). | 18 387 |
| 44 | behavio?r [change.mp](http://change.mp/). | 10 139 |
| 45 | models, nursing/ or models, organizational/ | 30 837 |
| 46 | or/19-45 | 1 591 897 |
| 47 | (model* or strateg* or intervention* or program*).mp. | 6 780 |
| 48 | 22 or 27 or 28 or 36 or 40 or 43 or 44 | 389 743 |
| 49 | 47 and 48 | 161 602 |
| 50 | 46 or 49 | 1 591 897 |
| 51 | 4 and 18 and 50 | 6 780 |
| 52 | Meta-Analysis as Topic/ | 17 942 |
| 53 | meta analy$.tw | 112 322 |
| 54 | metaanaly$.tw | 1 801 |
| 55 | Meta-Analysis/ | 97 340 |
| 56 | (systematic adj (review$1 or overview$1)).tw | 98 850 |
| 57 | Exp Review Literature as Topic/ | 10 551 |
| 58 | or/52-57 | 205 167 |
| 59 | cochrane.ab. | 52 749 |
| 60 | embase.ab. | 55 499 |
| 61 | (psychlit or psyclit).ab | 934 |
| 62 | (psychinfo or psycinfo).ab | 16 230 |
| 63 | (cinahl or cinahl).ab. | 17 468 |
| 64 | science citation index.ab. | 2 678 |
| 65 | bids.ab. | 441 |
| 66 | cancerlit.ab | 657 |
| 67 | or/59-66 | 87 674 |
| 68 | reference list$.ab | 14 276 |
| 69 | bibliography$.ab. | 14 552 |
| 70 | hand-search$.ab. | 5 304 |
| 71 | relevant journals.ab. | 989 |
| 72 | manual search$.ab. | 3 312 |
| 73 | or/68-72 | 34 473 |
| 74 | selection criteria.ab. | 26 460 |
| 75 | data extraction.ab. | 14 509 |
| 76 | 74 or 75 | 38 888 |
| 77 | Review/ | 2 429 942 |
| 78 | 76 and 77 | 28 024 |
| 79 | Comment/ | 717 040 |
| 80 | Letter/ | 1 003 380 |
| 81 | Editorial/ | 433 328 |
| 82 | animal/ | 6 687 808 |
| 83 | human/ | 18 317 354 |
| 84 | 82 not (82 and 83) | 4 770 502 |
| 85 | or/79-81,84 | 6 303 117 |
| 86 | 58 or 67 or 73 or 78 | 244 089 |
| 87 | 86 not 85 | 230 274 |
| 88 | randomi?ed controlled trial.pt | 513 947 |
| 89 | controlled clinical trial.pt. | 101 674 |
| 90 | randomi?ed.ti,ab. | 514 531 |
| 91 | placebo.ti,ab. | 198 627 |
| 92 | clinical trials as topic.sh. | 202 274 |
| 93 | randomly.ti,ab. | 272 141 |
| 94 | trial.ti. | 178 776 |
| 95 | or/88-94 | 1 191 787 |
| 96 | exp animals/not exp humans/ | 4 805 331 |
| 97 | 95 not 96 | 1 092 542 |
| 98 | 87 or 97 | 1 245 737 |
| 99 | 51 and 98 | 1 144 |
| 100 | Limit 99 to (english language and yr=”1994-Current”) | 1 112 |
