# Supplementary material for: Effectiveness of self-management support interventions for people with comorbid diabetes and chronic kidney disease: a systematic review and meta-analysis
Source: Syst Rev. 2018 Jun 13;7:84. doi: 10.1186/s13643-018-0748-z (PMC6001117; doi:10.1186/s13643-018-0748-z)
Supplement: Supplementary file 3 — Table S3. Characteristics of excluded studies (ordered alphabetically). (DOCX 12 kb) [file 13643_2018_748_MOESM3_ESM.docx]

**Table S3**. Characteristics of excluded studies (ordered alphabetically)

| **Study** | **Reason for exclusion** |
| --- | --- |
| Abdel-Kader 2009 | No response from authors to clarify study queries |
| Adair 2013 | No subgroup analysis to confirm right population |
| Adepoju 2014 | No subgroup analysis to confirm right population |
| Chao 2014 | No response from authors to clarify study queries |
| Chen 2011 | No response from authors to clarify study queries |
| Crowley 2013 | No subgroup analysis to confirm right population |
| Dansky 2003 | No subgroup analysis to confirm right population |
| De Brito-Ashurst 2012 | No subgroup analysis to confirm right population |
| Desroches 2013 | No subgroup analysis to confirm right population |
| Devins 2003 | No response from authors to clarify study queries |
| Drawz 2012 | No response from authors to clarify study queries |
| Eakin 2009 | No subgroup analysis to confirm right population |
| Flesher 2011 | No subgroup analysis to confirm right population |
| Foy 2011 | Not in relevant population |
| Glasgow 2012 | No response from authors to clarify study queries |
| Harris 1998 | No subgroup analysis to confirm right population |
| Holbrook 2009 | No subgroup analysis to confirm right population |
| Hung 2014 | Not an RCT (Commentary of RCT) |
| Isbel 2006 | No subgroup analysis to confirm right population |
| Ishani 2016 | No response from authors to clarify study queries |
| Leonardis 2012 | Study is incomplete |
| Lusignan 2013 | No subgroup analysis to confirm right population |
| Manns 2005 | No response from authors to clarify study queries |
| McCall 2011 | No response from authors to clarify study queries |
| Ong 2016 | No subgroup analysis to confirm right population |
| Peeters 2012 | No response from authors to clarify study queries |
| Piette 2000 | No response from authors to clarify study queries |
| Rifkin 2013 | No response from authors to clarify study queries |
| Selea 2011 | Not in relevant population |
| Sintchenko 2007 | Not in relevant population |
| Strand 2012 | Not in relevant population |
| Tricco 2012 | Not in relevant population |
| Walker 2013 | Not an RCT |
| Weber 2012 | No response from authors to clarify study queries |
| Wentzlaff 2011 | No response from authors to clarify study queries |
| Wong 2010 | No response from authors to clarify study queries |
| Yamagata 2016 | Study is incomplete |
