# Supplementary figures and images for: Effectiveness of self-management support interventions for people with comorbid diabetes and chronic kidney disease: a systematic review and meta-analysis
Source: Syst Rev. 2018 Jun 13;7:84. doi: 10.1186/s13643-018-0748-z (PMC6001117; doi:10.1186/s13643-018-0748-z)

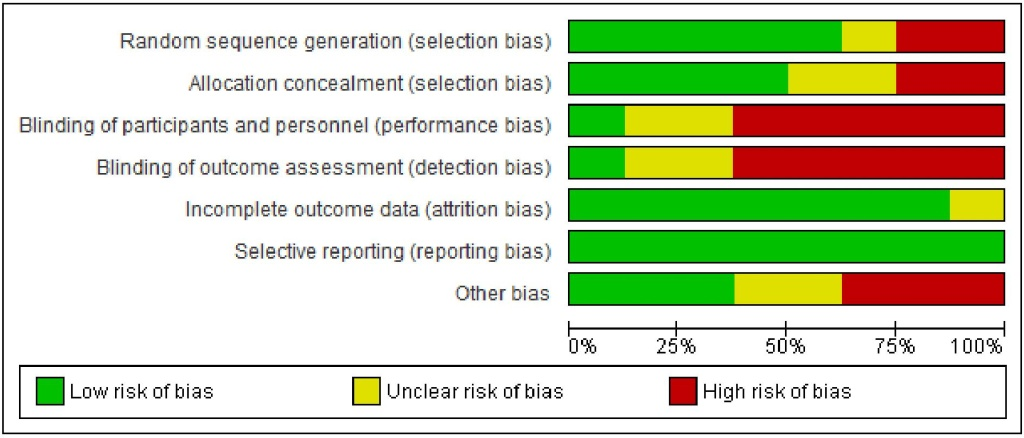

Supplement: Supplementary file 4 — Figure S1. Risk of bias: review authors' judgements about each risk of bias item presented as percentages across all included studies. (PNG 205 kb) [file 13643_2018_748_MOESM4_ESM.png]

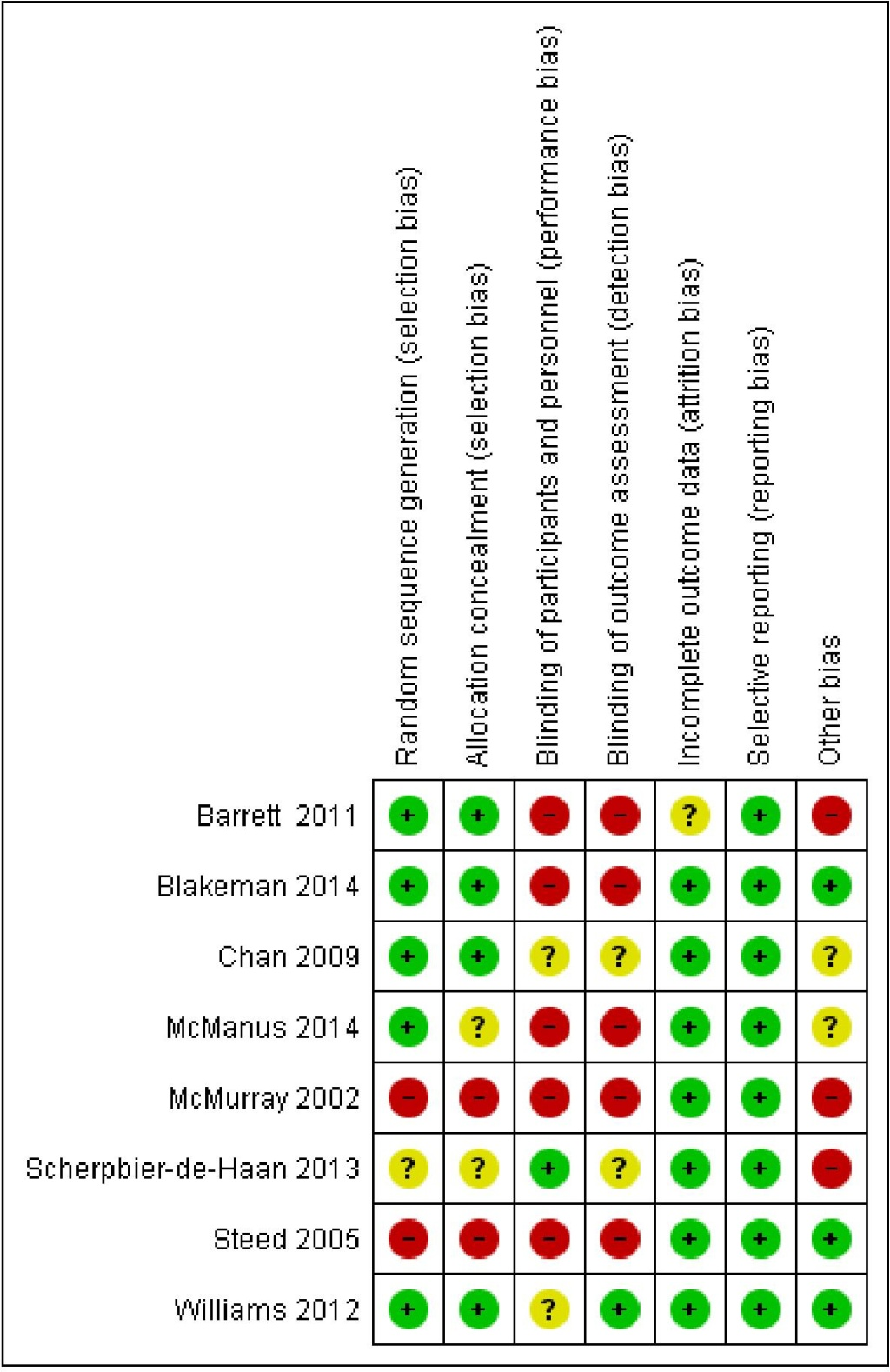

Supplement: Supplementary file 5 — Figure S2. Methodological quality summary: review authors' judgements about each methodological quality item for each included study. (PNG 583 kb) [file 13643_2018_748_MOESM5_ESM.png]
